# Body Composition and Energy Expenditure in Youth With Spina Bifida: Protocol for a Multisite, Cross-Sectional Study

**DOI:** 10.2196/52779

**Published:** 2024-07-02

**Authors:** Michele Polfuss, Kathryn Smith, Betsy Hopson, Andrea Moosreiner, Chiang-Ching Huang, Michele N Ravelli, Dan Ding, Zijian Huang, Brandon G Rocque, Rosemary White-Traut, Alexander Van Speybroeck, Kathleen J Sawin

**Affiliations:** 1 School of Nursing College of Health Professions and Sciences University of Wisconsin - Milwaukee Milwaukee, WI United States; 2 Department of Nursing Research and Evidence-Based Practice Children's Wisconsin Milwaukee, WI United States; 3 Department of Pediatrics USC Keck School of Medicine Children's Hospital of Los Angeles Los Angeles, CA United States; 4 Department of Mediciine University of Alabama at Birmingham Birmingham, AL United States; 5 Clinical and Translational Science Institute Medical College of Wisconsin Milwaukee, WI United States; 6 Zilber College of Public Health University of Wisconsin - Milwaukee Milwaukee, WI United States; 7 Biotechnology Center University of Wisconsin - Madison Madison, WI United States; 8 Department of Rehabilitation Science and Technology University of Pittsburgh Pittsburgh, PA United States; 9 Department of Neurosurgery University of Alabama at Birmingham Birmingham, AL United States; 10 College of Nursing University of Illinois at Chicago Chicago, IL United States; 11 Division of General Pediatrics USC Keck School of Medicine Children's Hospital of Los Angeles Los Angeles, CA United States

**Keywords:** obesity, overweight, body composition, energy expenditure, doubly labeled water, spina bifida, children, adolescents, wearable device

## Abstract

**Background:**

Obesity prevalence in youth with spina bifida is higher than in their typically developing peers. Obesity is associated with lifelong medical, psychological, and economic burdens. Successful prevention or treatment of obesity in individuals with spina bifida is compromised by (1) the lack of valid and reliable methods to identify body fat in a clinical setting and (2) limited data on energy expenditure that are necessary to provide daily caloric recommendations.

**Objective:**

The objectives of this study will be to develop 2 algorithms for use in youth with spina bifida in a clinical setting, one to model body fat and one to predict total daily energy expenditure. In addition, physical activity and dietary intake will be described for the sample.

**Methods:**

This multisite, prospective, national clinical study will enroll 232 youth with myelomeningocele aged 5 to 18 years (stratified by age and mobility). Participants will be enrolled for 1 week. Data obtained include 4 measures of body composition, up to 5 height measures, a ramped activity protocol, and a nutrition and physical activity screener. Participants will wear an accelerometer for the week. On the final study day, 2 samples of urine or saliva, which complete the doubly labeled water protocol, will be obtained. The analysis will include descriptive statistics, Bland-Altman plots, concordance correlation, and regression analysis.

**Results:**

The study received extramural federal funding in July 2019. Data collection was initiated in March 2020. As of April 2024, a total of 143 (female participants: n=76, 53.1%; male participants: n=67, 46.9%) out of 232 participants have been enrolled. Data collection is expected to continue throughout 2024. A no-cost extension until November 2025 will be requested for data analysis and dissemination of findings.

**Conclusions:**

This study furthers previous pilot work that confirmed the acceptability and feasibility of obtaining alternate height, body composition, and energy expenditure measures. The findings from this study will enhance screening, prevention, and treatment of abnormal weight status by facilitating the accurate identification of youths’ weight status category and recommendations of daily caloric needs for this population that is at higher risk of obesity. Furthermore, the findings have the potential to impact outcomes for youth diagnosed with disabilities other than spina bifida who experience similar challenges related to alterations in body composition or fat distribution or measurement challenges secondary to mobility issues or musculoskeletal problems.

**International Registered Report Identifier (IRRID):**

DERR1-10.2196/52779

## Introduction

### Background

#### Obesity in Spina Bifida

The prevalence of obesity is as high as 50% of youth and 64% of young adults with spina bifida (SB) [1]. Youth with obesity often remain obese as adults, increasing their risk of morbidity and mortality [2]. Obesity is associated with lifelong medical, economic, and psychological burdens [3-6].

SB is a birth defect involving an incomplete closure of the spine that occurs in 3.5 per 10,000 live births [7]. The higher incidence of youth with SB having obesity can be attributed to characteristics inherent in the condition, including (1) a lower percentage of lean mass and lower basal metabolic rate (BMR), (2) decreased function in the lower extremities that can alter the individual’s mobility dependent on the level of the spinal lesion, and (3) an overall reduced energy expenditure (although exact amounts are not quantified) [8-11]. Muscle hypoplasia and decreased growth and height velocity can further exacerbate the high percentage of body fat and is accentuated with the youth’s advancing age, higher level of lesion, and decreased mobility status [11-14]. Carrying excess body fat with SB increases the risk of decubitus ulcers, skin breakdown, social isolation and rejection, and surgical complications. It further decreases an individual’s mobility and the ability to self-manage their condition, undermines independence, and creates unnecessary barriers for caregivers who assist with daily care [15-18]. In addition, obesity creates the same risk of obesity-related comorbidities as in the typically developing (TD) population, including type 2 diabetes, metabolic syndrome, and cardiovascular disease [19-22], highlighting the need for obesity prevention. Thus, obesity in youth with SB places them in double jeopardy for poor physical health, creates additional barriers to independence, and can worsen the underlying conditions associated with the disability [18,23-27]. The focus on youth is important when addressing obesity-related issues because the prevention and treatment of obesity begin in childhood, when weight-related behaviors and habits are being formed and can promote successful weight management as the youth ages [19].

To address obesity in youth with disabilities, the Eunice Kennedy Shriver National Institute of Child Health and Human Development (NICHD) convened a group of experts in obesity and disabilities that identified barriers to healthy weight status in youth with disabilities and developed a research agenda to address the problem [28]. Two research priorities were identified: (1) the need for a clinically available, accurate measure of obesity; and (2) data on energy expenditure [28].

Specifically, the group recommended that the obesity measure to be developed be valid, reliable, and feasible for use in the clinical setting [28]. The group also highlighted the methodological concerns of using BMI in youth with disabilities, including the difficulties associated with accurately measuring the height of youth who primarily use wheelchairs and cannot stand independently and have inherent differences in body composition related to their diagnosis [28]. Accurate height measurement is an integral component of many body composition measures. The need for data related to energy expenditure was based on the dearth of information available specific to youth with disabilities and the lack of understanding of the impact that a physical disability has on youths’ energy requirements and nutrient needs [28].

Routine screening for obesity risk and documenting weight status is recommended for all youth and is integral for preventing and treating obesity and promoting health [19]. However, commonly used measures of body fat have been normed on the TD population [29] and often require measurement of height [30]. Individuals with SB have a decreased height velocity, atypical body habitus (eg, abnormal water and fat distribution and varying degrees of muscle atrophy), and orthopedic complications (eg, contractures, kyphosis, or scoliosis) that make it difficult to obtain an accurate height measurement [10,11,13,14,31-35]. The characteristics inherent in SB, along with measures normed on the TD population and reliance on the height measurement, lead to inaccurate body fat measurements or delayed identification of abnormal weight status for youth with SB.

#### Measurement of Body Composition

Proposals to modify the BMI [14,36] or use alternative measures of height or body composition to increase the accuracy of body fat assessment in SB have been made [10,12,30-32,37-42]. However, these modifications or alternative measures have yet to be tested in a sufficiently large sample, thereby limiting their application in this population. The benefits of BMI (eg, ease of use and cost-effectiveness) have prompted suggestions to adjust the BMI graph percentiles or obesity cutoff points for individuals with physical disabilities. One proposal suggested lowering the BMI graph percentiles by 50% for youth with higher-level lesions to improve obesity identification [14]. In 29 adolescents with physical disabilities (n=3 with SB), it was reported that using a more conservative cutoff point for obesity of 19 kg/m^2^ (female individuals) and 20 kg/m^2^ (male individuals) versus graph percentiles increased the classification accuracy [36]. However, these approaches have not been validated. The underlying need for an accurate height within a BMI calculation and other body composition measures has led to suggestions for using surrogate measures of height or length (eg, arm span, recumbent length, tibia height, and ulnar length) [14,30,43-45].

Beyond BMI, alternative body composition measures (eg, waist circumference, skinfolds, and bioelectrical impedance analysis [BIA]) that fulfill the requirements for being clinically available, cost-effective, and previously used in TD youth [46-52] or small samples of youth with SB [9,10,31,32,36,38,53] are promising but need further testing in larger samples. Alternate methods of estimating body fat with higher accuracy (eg, isotope dilution technique, dual x-ray absorptiometry [DXA], underwater weighing, and magnetic resonance imaging) are available [54,55] but are not easily accessible, cost-effective, or pragmatic for performing in a clinic setting or on an ongoing basis to monitor body weight trends [27].

#### Measurement of Energy Expenditure

Subsequent to the accurate identification of percentage of body fat, the need to provide anticipatory guidance on daily energy (kcal) requirements is essential to obesity prevention and treatment. Total energy expenditure is the combination of the individual’s BMR, thermic effect of food, physical activity energy expenditure, and the cost of growth in children [56]. The total daily energy expenditure (TDEE) is the average energy expended in a 24-hour period. Weight management strategies are based on the overly simplified premise of energy balance (ie, energy in [nutritional intake] must equal energy out [energy expenditure] to maintain weight [57]). When there is a balance deficit, a change in weight will occur (eg, energy in>energy out=weight gain). Although more complex than this, knowledge of TDEE is essential to accurately guide energy requirements specific to daily caloric intake.

In the TD population, estimated energy requirement equations based on the child’s sex, height, and weight are used to determine average calorie needs per day [58]. Unfortunately, these equations are based on the metabolic rate and reference height and weights of TD youth, which are not applicable for youth with SB. In fact, the use of these equations would grossly overestimate the energy needs of youth with SB, resulting in overfeeding and further weight gain [34]. There is evidence supporting that youth with SB have a reduced energy expenditure, but the amount has not been specified [8,31,32,34,59]. Similar to body composition measurement, preliminary studies in small samples have been conducted but need to be tested in larger samples with validated criterion measures of energy expenditure [31,32,34]. Evidence-based information on TDEE is key to future research endeavors, including creating and testing interventions for weight management.

Stable isotope techniques are reliable methods for measuring energy expenditure and body composition within a 2-compartment model comprising fat-free mass (FFM) and fat mass (FM). The doubly labeled water (DLW) technique is widely recognized as a gold-standard tool for measuring TDEE [60]. This method involves the ingestion of water containing 2 stable isotopes, deuterium (^2^H) and oxygen-18 (^18^O), which act as tracers when ingested. The dosage of DLW is determined based on the individual’s body weight. The analysis is performed on the individual’s body fluids (saliva, blood, or urine) through isotope ratio mass spectrometry to measure the elimination of the tracers over a specific time frame [60]. The difference in the elimination rate of the tracers from the body allows for the calculation of carbon dioxide production, a product of energy metabolism that is used to compute TDEE [60]. Furthermore, the added tracers can also be used to estimate the individual’s total body water (TBW) through the isotope dilution technique. Equations are typically applied to DLW-derived TBW to determine body fat percentage, an age-related adjustment for chemical maturation of FFM.

#### Pilot Studies

This study furthers previous pilot work that examined the feasibility of obtaining multiple height and body composition measures and then used these measures to generate preliminary models to predict FM as measured using a DXA scan in 15 youths aged 4 to 18 years with SB. A total of 4 measures of height (segmental, arm span, recumbent, and standing height [if able]) and 5 body composition measures (Bod Pod, 7-site skinfold, traditional BMI calculation, BIA, and DXA) were found to be feasible to obtain and generally accepted by participants [61]. A second pilot study examined the feasibility of completing different measures of energy expenditure and activity using a metabolic cart, portable calorimeter, accelerometers, and DLW in 36 youths with SB (n=18; 9 ambulatory and 9 who primarily used a wheelchair for mobility) and Down syndrome (n=9) and a control group of TD youth (n=9) without chronic illness. DLW analysis was found to be feasible (34/36, 94% of participants completed the protocol), and accelerometers were worn without concern. The DLW used urine and saliva as samples, with each being feasible and acceptable. Results showed that, when matched for FFM, the recommended daily caloric intake based on TDEE averaged 800 calories less per day for the participants with SB who primarily used a wheelchair (*P*=.001) as compared to the controls [62].

The objective of this study was to provide a method of measurement that accurately assesses body fat and the estimation of energy expenditure in youth with SB in a clinical setting.

### Study Aims

This national study is convening a sample of 232 youths with SB to complete the following aims.

#### Aim 1

The first aim is to develop an algorithm that accurately models body fat percentage and categorizes weight status in youth with SB using clinically feasible, cost-effective, and efficient measures of body composition (4-site skinfolds, waist circumference, and BIA) or surrogate height measures (arm span, recumbent, knee height, and ulnar length). The subaim is to examine whether mobility status, sex, BMI using surrogate height, and pubertal status are important determinants in the model and determine whether corrections are needed for these variables.

#### Aim 2

The second aim is to measure TDEE using DLW and develop a prediction equation for the energy requirements of the sample. The first subaim is to examine average TDEE based on age, sex, and mobility status (walks or primarily uses a wheelchair), and the second subaim is to develop an equation based on actual TDEE to predict energy requirements using a best-fit model based on FFM, sex, age, mobility status, assistive device use, and height or weight. The exploratory aim is to describe the dietary intake and the physical activity frequency and duration in youth with SB using a nutrition screener, physical activity screener, and accelerometers. This exploratory aim provides a preliminary understanding of obesity-related behaviors, which is currently lacking for this population.

## Methods

### Study Design

A multisite, prospective cross-sectional study of youth with the most severe form of SB, myelomeningocele, is in progress. The project is being conducted collaboratively through 4 regional pediatric SB programs.

### Setting

The SB programs from across the country were strategically chosen based on (1) the ethnic and geographic representation of clinic patients, (2) broad research experience with this vulnerable population, (3) participation in the Centers for Disease Control and Prevention National Spina Bifida Patient Registry (NSBPR), and (4) the ability to recruit the needed sample size. After the COVID-19 pandemic, during the initial implementation phase of the protocol, one of the original sites withdrew after data collection challenges and was replaced by an alternate site. In total, 3 of the 4 sites use their associated clinical and translational science institutes’ research unit for study visits, and the fourth uses their designated clinical research setting. Each site has a predetermined recruitment goal based on its patient enrollment. The NSBPR investigators at each site have established routine communication patterns, annual meetings, and a history of collaboration to determine, gather, and evaluate data collected through the registry. Each site recruits and collects data on its predetermined number of participants and has coinvestigators and staff to support their roles.

### Sample Characteristics

On the basis of the data from the NSBPR [7], we estimated that 1399 youth are seen yearly in the 4 participating SB programs. Among these, we expect approximately 81%, or 1133, to be diagnosed with myelomeningocele. Of those, 725 (64%) are expected to be aged between 5 and 18 years and eligible for recruitment. On the basis of the NSBPR data [7], we expect our sample to be approximately 52% female.

This study has a targeted enrollment of 232 youths (aged 5-18 years) diagnosed with myelomeningocele-type SB. Purposive sampling is being used to achieve a minimum of 60 youths who identify as being Hispanic or Latino and 116 who primarily use a wheelchair for mobility. Participants are stratified by age (5-11 years and 12-18 years) and mobility status (ambulates independently vs primarily uses a wheelchair for mobility) to increase the usefulness of the study findings to both mobility groups (Table 1). Once the collection is complete for a subgroup, sites will be notified to stop recruiting for that specific mobility status, age, or ethnicity.

**Table 1:** Sample stratification (N=232).

	Ambulatory			Wheelchair	
	Sample, n (%)	Minimum Hispanic, n (%)	Sample, n (%)		Minimum Hispanic, n (%)
Aged 5-11 years	58 (25)	15 (6.5)	58 (25)		15 (6.5)
Aged 12-18 years	58 (25)	15 (6.5)	58 (25)		15 (6.5)
Total	116 (50)	30 (12.9)	116 (50)		30 (12.9)

The chosen age range (5-18 years) allows for representation of 2 developmental stages that include prepubertal and pubertal status; children in these groups were successful at following the protocol during the pilot study. In addition, the initial age of 5 years was chosen based on the likelihood of being able to cooperate with the study protocol. The end cutoff point of 18 years was chosen because many youth transfer to an adult SB program at that time.

The specific inclusion of minority groups provides a more accurate representation of the population of youth with SB and makes the findings more useful as obesity prevalence has been documented as varying by ethnic descent [3]. To accommodate participants who do not speak English, interpreters are sufficiently budgeted for, and all study materials are professionally translated into Spanish.

### Inclusion and Exclusion Criteria

The inclusion criteria are (1) youth aged between 5 and 18 years diagnosed with myelomeningocele and (2) English or Spanish speakers (interpreters will be provided).

Participants are excluded from the study if they meet one or more of the following criteria that either interfere with their ability to complete the protocol or compromise the study results: (1) a medical restriction to a 6-hour fast, (2) pregnancy, (3) expectation to participate in abnormally vigorous and out-of-the-ordinary activity during the 7-day test period, (4) living or traveling >200 miles from the study site during the week before or during the protocol, (5) use of supplemental oxygen, (6) a pacemaker, (7) blood transfusion or intravenous infusion of >500 mL of intravenous fluids the week before the test date or expectation to do so during the 7-day test period, (8) claustrophobia or predisposition to the sensation of claustrophobia (for sites including a resting metabolic cart assessment), and (9) an active viral or bacterial illness during the initiation of the 7-day protocol (if study participants are ill during their in-person study visit, they are rescheduled).

### Recruitment

Recruitment is conducted by the participating pediatric SB programs, their surrounding communities, and national organizations (eg, Spina Bifida Association) through newsletters, social media, and referrals from family members and health care providers. It is reiterated during and within the consent and assent process that participation is completely voluntary and that there will be no consequences if individuals do not take part or complete the study.

Recruitment processes used in the previous pilot studies were effective in achieving targeted participation and are being replicated. Recruiting strategies include institutional review board (IRB)–approved advertisements that are posted and distributed within clinics, locations where families congregate, and special events that focus on families with disabilities. In addition, the advertisements can be mailed to patients enrolled in the participating SB programs, shared through social media sites such as X (formerly known as Twitter) and Facebook, and posted on the websites of relevant professional organizations. Within the clinic, a Permission to Contact Form is used, which allows a family (if interested) to indicate where, how, and when they would like to be contacted to discuss the study further. Recruitment materials include a pictorial orientation study manual (in print and web-based), modified from that of our initial pilot study, explaining the study protocol and measurements so that families can make an informed decision to participate. By using SB programs as our recruitment sites, families that have an ongoing relationship with the program can participate in this study in a familiar environment.

### Participant Reimbursement

To reimburse the families for their time and cost of participation in the study, each family is offered a US $100 gift card for completing the first-day (<5 hours) appointment and a US $150 gift card when the day-7 urine specimens and accelerometers are returned to the study site. The second gift card is sent only after the day-7 samples are received. All supplies and prepaid shipping mailers are provided to the family before their departure on day 1. Early in the implementation process, unanticipated transportation and childcare issues were encountered by a subset of sites. Due to this concern, an amendment was submitted to allow for an additional voucher of up to US $125 per the site’s discretion to accommodate families who expressed interest in participating in the study but acknowledged barriers (eg, lack of transportation, distance to the clinic requiring a hotel stay for an early-morning visit necessary due to fasting for the child, or childcare), along with local parking vouchers if relevant. The request and reason for use are documented by each site. Study dates are on weekdays and include flexible appointment times to meet the needs of the families. Morning appointments are recommended based on the need for a minimum 6-hour fasting status before testing.

### Retention Strategies

Retention of study participants (to complete the full 7-day protocol) is enhanced by replicating strategies successfully used in our pilot studies, including the provision of (1) a comprehensive explanation of what the study entails (aided by the pictorial recruitment manual) so that the family can make an informed choice to participate, (2) flexible scheduling options, (3) clear instructions provided verbally and in print when they leave on day 1, and (4) all materials needed for the collection and the return of the day-7 urine samples and accelerometers with prepaid mailing labels and supplies. To enhance retention and completion of data collection, a call or SMS text message is made or sent to the family on day 5 or 6 to remind them of the final samples needed on day 7 and review instructions and answer questions. The final retention aid is that the second gift card is not sent to the family until the day-7 sample collections are received. The process and requirements for receipt of the second gift card are discussed during their day-1 visit, which we believe may support study retention.

### Measures and Instruments

Table 2 outlines vital signs and anthropometrics, Textbox 1 provides a listing of body composition and energy expenditure measures, and Textbox 2 provides study instruments. All instruments and study materials are available in English and Spanish. Specific training, orientation, and validation checks are completed for data collectors along with the provision of a study manual with detailed instructions supported with pictures. For all measures, a standardized process is followed to obtain the measure, and an open comment box is available for the data collector to provide additional details related to any challenges encountered (eg, orthopedic complications) to be considered during analysis. In total, 2 of the measures, DXA and resting metabolic rate through a resting metabolic cart, are only being collected at the lead principal investigator’s site to provide a subset of participants to be examined as a secondary validation assessment for the DLW. The decision to use this site for these additional measures was made as the measures were successfully collected during the pilot work at this site.

**Table 2:** Vital signs and anthropometrics.

Measure		Procedural operations
Vital signs		Baseline heart rate, blood pressure, and respiratory rate are obtained.
**Weight (measured to the nearest 0.1 kg)**		
	Body weight	Participant removes shoes, extra clothing (jackets), and assistive devices and is weighed on a medical-grade calibrated standing or wheelchair scale. After, the wheelchair is weighed separately and subtracted from the total weight of participant and wheelchair. If braces are needed to safely stand, the youth is weighed with the braces on (total weight), and then the braces are removed and weighed alone. The weight of the braces is subtracted from the total weight to obtain the final youth weight.
**Height (measured to the nearest 0.1 cm)**		
	Standing	If able to stand independently and safely, the participant removes shoes or assistive devices and stands erect to the best of their ability with their back and heels against a wall-mounted stadiometer. The stadiometer arm is brought to the top of the youth’s head.
	Arm span	Participant abducts shoulders 90° and fully extends arms and hands. A flexible tape measure is placed posteriorly from the tip of the middle finger to the tip of the opposite middle finger with staff on each side to assist with outstretching of arms perpendicular to the body. Each measure is obtained 3 times, and the average is used.
	Recumbent length	Participant lies supine on a length board on a flat surface. The measure is taken from the crown of the head to the base of the feet. Staff assists with leg extension or ankle dorsiflexion if there are orthopedic issues. Each measure is obtained 3 times, and the average is used.
	Knee height	With the participant seated with the knee at a 90° angle, the fixed blade of the caliper is placed at the distal end of the calcaneus and the sliding blade is placed on the anterior side of the distal condyle of the femur, with the caliper shaft aligned with the tibia [30]. Each measure is obtained 3 times, and the average is used.
	Ulnar length	While the participant is seated, the forearm rests on the table with the palm down, fingers extended, and elbow bent 90° to 110°. A measure is taken from the elbow (olecranon process) to the wrist (styloid process) with an anthropometer or measuring tape [44]. Each measure is obtained 3 times, and the average is used.

**Body composition and energy expenditure assessment.**

**Measure and procedural process**

- Waist circumference: measure at 2 different landmarks—the umbilicus and immediately above the right iliac crest at the midaxillary line—using a flexible tape measure. Measures are obtained in recumbent position and standing (if able to stand) and to the nearest 0.1 cm 3 times, and the average is used.
- Bioelectrical impedance analysis (BIA): confirm no vigorous physical activity, food, or substantial drink for a minimum of 3 hours. Using an InBody S10 BIA machine, place electrodes on the youth’s extremities and have them sit for 10 minutes before the first measure. Manually input the youth’s height and weight and then obtain the measure (approximately 40 seconds to complete). Repeat assessment using the average of each height measure (standing, arm span, recumbent, knee height, and ulnar length).
- 4-site skinfolds: skinfold calipers are used to pinch fat at 4 body landmarks (triceps, biceps, suprailiac, and subscapular). Each skinfold measure is obtained 3 times, and the average is used.
- Doubly labeled water (DLW): verify a minimum of 6 hours of fasting or light fluid ingestion. Approximately 1-3 oz of DLW (dosed on body weight) are consumed. After drinking the DLW, 50 mL of drinking water are added to the empty bottle and consumed by the youth. On day 1, urine or saliva specimens are collected at baseline (before DLW) and hours 1 (discarded), 3 (kept), and 4 (kept) after consuming DLW. The family collects 2 urine or saliva specimens 1 hour apart on day 7 with the provided supplies and mails the specimens back to the study site.
- Dual-energy x-ray absorptiometry (DXA; only site 1): before the scan, female participants are screened for risk of pregnancy per hospital policy. If there is risk of pregnancy, the participant does not undergo DXA scan and continues with the remainder of the protocol. If the study participant has metal implants, they are excluded from the DXA scan as this is a contraindication. External artifacts are removed (eg, buttons, zippers, and jewelry). Hospital scrubs will be available if needed. With the participant lying supine on the open DXA table, the scan arm passes over the participant’s body in a series of transverse scans from head to toe. If there is no risk of pregnancy in the parent, they are allowed to remain with the youth during the scan but asked to remain 1 meter away from the scanner.
- Resting metabolic cart (only site 1): this noninvasive test uses indirect calorimetry to measure resting metabolic rate [63]. This portable unit measures the concentration of oxygen and carbon dioxide in air streams entering and exiting a clear canopy tented over the youth. The participant lies quietly for 30 minutes before and during the test while respiratory gas exchange is measured and averaged. The youth is able to watch television with their parent from the bed with external distractions (eg, noise and lights) minimized.
- Accelerometer—measured physical activity: physical activity is measured using the ActiGraph GT3XP-BT accelerometer (ActiGraph LLC) provided on day 1 to be worn for the following 7 days on the participant’s nondominant wrist and removed during water activities (eg, swimming and bathing) [64-67]. Raw data at 30 Hz are collected and converted to counts for analysis. During day-1 testing, participants perform a timed ramped protocol based on the individual’s perceived exertion at baseline, light, moderate, and vigorous intensity levels aided by a validated pictorial perceived exertion chart that commiserates with their mobility status (eg, wheelchair or walking) [68].
- Heart rate monitor: heart rate during the timed ramped protocol is recorded using an electrocardiogram-based heart rate monitor (Polar H7; Polar Electro Inc) mounted on the participants’ chest with an elastic band. Recorded heart rate is time stamp synced with the accelerometer data to provide a reference of measured physical activity intensity.
- Fat-free mass (FFM): FFM (kg) = total body water (TBW; kg)/hydration factor of FFM based on the child’s age and sex [69]. FFM comprises all body components except fat or adipose tissue [54]. FFM contains 73.2% water in healthy adults, but in children, the hydration of FFM is higher and is based on age and sex as listed in the study by Fomon et al [70].
- Fat percentage: fat percentage=100 – (body weight – [TBW/hydration factor of FFM]). The hydration factor is based on the child’s age and sex [69]. Fat percentage is defined as the percentage of body weight that is fat. The equation uses the hydration factor of FFM for children in the work by Fomon et al [70].
- TBW: TBW = ([N_O_/1.007] + [N_D_/1.043])/2, where N_i_= ([W × A/a] × [ΔDD/ΔBW])/1000 – cumulative fluid intake (kg) [60]. The water content of the body at birth is 70% to 75% but decreases in adulthood. Water (intra- and extracellular) is contained exclusively within the FFM [60]. The TBW is determined by measuring and adjusting the dilution space of oxygen-18 (N_O_) and deuterium (N_D_) in the body water pool using nonaqueous exchange values of 1.007 and 1.043 for each isotope, respectively. In the calculation, N_i_ is the dilution space for each isotope, “W” is the amount of water used to prepare the diluted dose, and “a” is the amount of isotopes used in the dilution. “A” is the dose of DLW consumed by the participant, ΔDD is the isotope enrichment measured in diluted dose, and “ΔBW” is the plateau enrichment measured in body water based on day 0 [60].
- Total energy expenditure (TEE) and total daily energy expenditure (TDEE): TEE (kcal/day) = rCO_2_x (1.106 + 3.94/RQ). TEE is the total energy used by our body to maintain life [71], including basal metabolic rate, the thermic effect of food, and physical activity (and growth in children) [56]. In the TEE calculation, the rate of carbon dioxide production (rCO_2_) is based on the body’s elimination of isotopes (rCO_2_= 0.4554 × TBW × [(1.007 × k_O_) – (1.043 × k_D_)] × 22.26, in which 22.26 is the gas constant for carbon dioxide in L/mol), and RQ is the respiratory quotient estimated from typical diet [72]. In our study, TEE is measured over 7 days and averaged to obtain the TDEE.

**Instruments.**

**Pubertal status**

- Youth pubertal status by parent report is based on a pictorial and written definition of Tanner stages 1 to 5 (development of external genitalia for male individuals and breast development for female individuals).
- Tanner stage 1=prepubertal and stages 2 to 5=pubertal. The purpose for this study is not specificity of stage but to discern prepubertal vs pubertal stages. Parent report of youth was valid when compared to health care provider assessment (*r*=0.75 to *r*=0.87; P≤.001;*k*=0.13-0.55) [73].

**Screener—measured nutrition**

- Block Kids Food Screener (version 2; NutritionQuest, 2007). The screener is self-administered to the child with parent assistance, as needed. The screener takes approximately 10 to 12 minutes to complete [74]. This 41-item screening instrument assesses intake by food group by amount (3-point scale) and frequency (6-point scale) consumed in the last 7 days. When compared to 24-hour dietary recalls, they were correlated between 0.526 for vegetables and 0.878 for potatoes. Bland-Altman plots indicated no systematic difference between the 2 based on food groups [75].

**Function**

- Pediatric Evaluation of Disability Inventory–Computerized Adaptive Test (PEDI-CAT; CREcare, LLC; 2007). The PEDI-CAT measures abilities in the ages of 2 to 21 years in 3 functional domains—daily activities, mobility, and social or cognitive—and a responsibility domain. Uses the item response theory statistical model to estimate the ability from a minimal number of most relevant items. It has positive reliability (intraclass correlation coefficient=0.96-0.99) and discriminant validity between children with and without disabilities (P<.001) and a mean completion time of 12.66 (SD 4.47) minutes [76].

**Screener—measured physical activity**

- Block Kids Physical Activity Screener (NutritionQuest, 2003). This 9-item screening tool assesses the frequency (6-point scale) and duration (4-point scale) of physical and sedentary activity in the last 7 days. The tool was validated for children aged 8 to 17 years, so it is not used with our participants aged 5 to 7 years. It takes approximately 5 minutes to complete.
- When used to estimate kilocalories based on weight, age, intensity, frequency, and duration in 48 children against an accelerometer, partial correlations (controlling for age and weight) were significant (r=0.56; P<.001) [77].
- Without a validated measure of physical activity for youth with disabilities, special considerations are taken with this instrument, and accelerometers are used to obtain an objective measure of activity. Participants are directed to substitute wheelchair options in the questions (ie, substitute wheeling for walking). We added 1 question asking youth to share any activities performed in the last 7 days that were otherwise not captured.

**Demographic form (parent completed)**

- Youth demographics include age (month and year), ethnicity, gender, level of education, participation in school health service plan, level of lesion, mobility status, assistive devices used for mobility, and concurrent diagnoses.
- Parental or legal guardian demographics include age, marital status, relationship to child, family income, educational level, ethnicity, and work status.

**Post–day 1 survey**

- Parents complete questions related to measurements performed during day 1, weight management in children with spina bifida, stress, and quality of life. The child (aged ≥12 years) completes 3 questions related to quality of life.

**Patient-Reported Outcomes Measurement Information System (PROMIS) parent-proxy peer relationships**

- A validated PROMIS questionnaire that includes 7 questions related to the child’s peer relationships is answered by the parent using a 5-point scale from never to almost always.

**Accelerometer and sample collection log**

- A 2-page log is provided to the family before leaving the day-1 visit. This log allows for the documentation of when the accelerometer was not worn and why, any unusual notes (eg, illness or unusual activities), and reminders for the day-7 urine or saliva sample collections.

### Procedures

After agreeing to participate (see the *Recruitment* section), consenting and assenting is completed, and a 5-hour morning appointment on day 1 is scheduled. A reminder call is placed to the family before the visit to review the study protocol and fasting requirements and answer any questions. On the day of the visit, the fasting status of the child is confirmed, and standard-of-care vital signs are obtained, including a baseline heart rate, blood pressure, and respiratory rate. The youth’s weight (wheelchair or standing based on mobility status) is obtained and used to verify the accurate dosing of DLW.

A baseline urine or saliva sample (participant’s choice) is obtained. The method of urine collection depends on the youth’s home bladder regimen. If they typically perform clean intermittent catheterization, catheters are provided for them (or their parent) to collect urine; if they typically void, a clean catch in a clean urine hat is completed. If they prefer to provide a saliva sample, they are asked to collect 2 to 3 mL of saliva in a tube.

After baseline urine or saliva sample collection and measurement of weight, DLW is dosed based on the youth’s weight and provided to the youth (1-3 oz depending on the youth’s weight) in a sterilized bottle provided by the laboratory and requested to be consumed using a straw. After drinking the DLW, 50 mL of drinking water are added to the empty DLW bottle and swished around, and the youth is asked to drink the added water to ensure that the DLW was completely consumed. Subsequent urine and saliva collection are conducted at hours 1 (only urine), 3, and 4 after consumption of DLW. The hour-1 sample is collected but discarded as the urine is not isotopically equilibrated with body water at this time and no saliva is collected at this time. The hour-3 and hour-4 samples are collected, timed, and saved for analysis. After hour 1 or between hours 1 and 3, the youth is offered a small snack that is consistent with the caloric requirements (≤250 kcal) of the DLW protocol, such as a granola bar, prepackaged snack, or small can of PediaSure supplement, and up to 8 oz of water.

Between timed urine or saliva samples, up to 5 measures of height (stadiometer [if able to stand], arm span [adjusted by level of lesion], recumbent length, knee height, and ulnar length) and 3 additional measures of alternative body composition beyond DLW (waist circumference, 4-site skinfold, and BIA) are collected. In addition, for participants at site 1, the child has a resting metabolic cart and a DXA scan performed in the hospital’s radiology department if no contraindications are present (eg, metal implants in the body). The resting metabolic cart measurement is completed as the resting metabolic rate is the largest contributor to TDEE. The DXA scan is used to address potential age-related variation in bone mass and hydration and provides coefficients between the DXA and TBW, eliminating concern of variance [69]. All sites conduct a ramped protocol while the study participant wears an accelerometer on their nondominant wrist and a heart rate monitor. The ramped protocol includes walking or wheeling in their wheelchair (based on ambulatory status) for set periods at different levels of intensity (eg, sedentary, light, and moderate to vigorous). Activities include 5 minutes each for sitting quietly and playing with Legos for arm movement, walking or wheeling slowly, and walking or wheeling at a normal pace. The final activity includes walking or wheeling at a fast pace for 6 minutes. After each activity, the child is asked to rate their perceived level of exertion using a pictorial scale [68].

Parents complete the demographic form, including a report of the youth’s Tanner stage, a post–day 1 survey that includes questions on the measurements performed and weight management in children with SB, the Patient Reported Outcomes Measurement Information System parent-proxy peer relationships questionnaire to assess their child’s peer relationships, and the Pediatric Evaluation of Disability Inventory–Computerized Adaptive Test survey related to their child’s function. Youths, with parental assistance as needed, complete the nutrition screener and physical activity screener. The physical activity screener was developed for youths aged 8 to 18 years, so youths aged between 5 and 7 years will not be assessed using this instrument.

Before the end of the day-1 appointment, the family receives a “participant kit” containing all materials needed for urine or saliva collection at home: sterile urine cups and urine hats or saliva collection tubes, labels with participant ID number and space for time and date of collection to be completed, and appropriate-sized catheter if the participant uses it. In addition, the participants are asked to continue wearing the accelerometer provided on their nondominant wrist for the following 7 days. They are provided an accelerometer wear and sample collection log to document when and why the accelerometer was removed and reminders for day-7 urine or saliva sample collection. At the end of the day-1 visit, the family receives a US $100 gift card in return for their time and travel, a US $25 hospital cafeteria voucher, and any additional site-specific gift cards (eg, parking or travel).

On day 7, the participants are asked to collect 2 urine or saliva samples 1 hour apart as close to their original appointment time of day as possible. Families keep urine or saliva samples refrigerated until they are able to mail them back along with their accelerometer to their original collection site using prepaid and addressed mailers and packing materials that are provided. After the study team receives the final samples, the family is mailed their second and final gift card for US $150. The gift card at the end of the study is emailed or mailed based on the participants’ preference. If mailed, the cards are sent through the US Postal Service and require a signed signature for receipt of mail. Table 3 provides a summary of the protocol.

**Table 3:** Study protocol overview.

Study date			Description
**Before day-1 study date**			
	Recruitment	Recruit participants and obtain consent and assent. Study team members complete enrollment questions as to their assessment of whether the recruited participant enrolled in the study or chose not to and the possible reasons.	
	Preparation	Confirm appointment date and time, fasting requirements, no active illness, catheter size, and estimate of youth body weight.	
**Day 1—appointment day with participant**			
	Day-1 study visit	Consent and assent, if not completed during recruitment. Obtain body weight × 1. Obtain pre-DLW^a^ baseline urine or saliva sample. Sample is timed and stored. Provide DLW based on body weight. After DLW consumption, obtain urine or saliva at hour 1 (discard), hour 3 (time and store), and hour 4 (time and store). Between urine or saliva collections, obtain height, body composition, and additional measures: Arm span, recumbent length, ulnar length, knee height, and stadiometer (if able to stand; each × 3) are obtained. Waist circumference and skinfold measures (each × 3); bioelectrical impedance analysis with each height measure are obtained. Vital signs (heart rate, blood pressure, and respiratory rate) are obtained. Ramped protocol of perceived exertion while wearing an accelerometer and heart rate monitor are obtained. Resting metabolic cart is obtained at 1 site. DXA^b^ scan is obtained at 1 site if no medical contraindications are present. Snack: Provide snack option (meal replacement drink [PediaSure] or prepackaged snack [≤240 kcal] and ≤8 oz of water) to the participant. Surveys: Parents complete demographics, PEDI-CAT^c^, post–day 1 survey, and Tanner stage of youth questions. Youth aged 8-18 years (with parent assistance as needed complete the Block Kids Physical Activity Screener. Youth aged 5-18 years (with parent assistance as needed) complete the Block Kids Food Screener. Before leaving the clinic: Provide the family with the Participant Kit; review materials and day-7 urine or saliva collection, mailing instructions, and research team contact information. Remove the heart rate monitor. Review the use of the accelerometer to be worn on the nondominant wrist for 7 days except when bathing, swimming, or showering. Provide a US $100 gift card, a US $25 cafeteria gift card, and any additional site-specific gift cards to the family for day 1.	
Day 5 and 6			Call or SMS text message to family to remind them to collect day-7 urine samples and review mailing instructions for urine or saliva samples and accelerometers to be mailed back to the collection site.
Day 7			Family collects 2 urine or saliva samples and mails back samples and accelerometer.
After day 7			Upon receipt, confirm obtaining samples and accelerometer. Disinfect the accelerometer; process samples; and complete study documentation, including notification that the final gift card can be sent to the family.

^a^DLW: doubly labeled water.

^b^DXA: dual x-ray absorptiometry.

^c^PEDI-CAT: Pediatric Evaluation of Disability Inventory–Computerized Adaptive Test.

### Data Analysis

Descriptive statistics, including mean, SD, median, and range, will be calculated for each variable or measurement. When analyzing arm span, knee height, ulnar length, waist circumference, and skinfolds, additional analysis or equations will be used based on the literature [30,38-41,43-48,50-52,78,79]. For BMI (kg/m^2^) and bioelectrical impedance measures, the average of each surrogate height measure will be used. Arm span can be used as a direct measure of height if there is no leg muscle mass loss (low-level lesions), or it can be modified if there are higher-level lesions and variations in leg muscle mass. If there is partial leg muscle loss (midlevel lesions), arm span × 0.95 can be used; with high-level lesions and complete leg muscle loss (thoracic-level lesion), arm span × 0.90 will be used. We will consider use of the measured arm span with and without the adjustment based on the level of lesion for our analysis. For knee height, 3 prediction equations developed for use with individuals with mobility impairment (cerebral palsy and Duchenne muscular dystrophy) [43-45] will be used. For ulnar length, a prediction equation using the ulnar length and age of the youth will be used [44].

For aim 1, Bland-Altman plot analysis and concordance correlation will evaluate the agreement between DLW body fat percentage and modified BMI, waist circumference, BIA, and skinfold measures. This marginal association analysis will also provide information for the relationship between different measures (linear, quadratic, and nonlinear association). To construct an algorithm that can predict body fat percentage, we will first choose the measures of skinfold thickness at 4 sites in our base model using the equations by Jackson and Pollock [79], Slaughter et al [51], and Gurka et al [78] that have been successfully applied to several adolescent populations [80]. To further improve accuracy, we will construct linear gender-specific regression models by considering the modified BMI (eg, weight or arm span and weight or ulnar length), FFM, waist circumference, mobility status, race, age, or pubertal status as potential predictor variables. Stepwise linear regression and all-possible-subsets regression procedures will be used to develop prediction equations. Potential interactions between age, race, and other variables will be examined. The Mallow Cp statistic and the Schwarz-Bayesian criterion will be used to select the appropriate models. The resulting predicted body fat percentage is a weighted sum of a subset of those potential predictors, where the weights are the parameter estimates associated with each predictor in the regression model. Predictive accuracy will be evaluated using the mean square error through a 10-fold cross-validation; all the samples will be randomly split into 10 parts, 9 folds of the samples will be used to build the regression model, and the remaining hold-out samples will be used to test the predictive accuracy between the predicted and DLW body fat percentage. This procedure will be repeated until all the samples are tested. Upon completion of our body fat percentage algorithm, we can use the distribution of this metric to categorize weight status (obesity, overweight, and normal weight) by selecting appropriate cutoff points. The determination of cutoff points to use for the categorization of the weight status was thoughtful and deliberate. While there are no accepted cutoff points for body fat percentage to define obesity or other weight categories for youth, we will use cutoff points based on body fat percentages correlated to BMI percentiles presented by McCarthy et al [81] that are age specific to account for the growth and development of the child. We will use the agreed-upon criteria for categorizing weight status: body fat percentage between 85% and 94% for overweight, ≥95% for obesity, and 5% to 84% for normal weight [19].

For aim 2, we will predict TDEE by estimating BMR and physical activity level and using these as our primary predictors. Several BMR equations from cross-sectional studies with good model performance (ie, *R*^2^=0.7-0.8) have been reported [82] that mainly used age, FFM, FM, and weight as key predictors. Thus, we will use those as our base model predictors and further consider height, race, gender, assistive devices used in ambulation, pubertal status, and mobility as potential predictors. Furthermore, the subset of 42 participants who will undergo a resting metabolic cart to measure resting energy expenditure will provide additional data to construct an equation for predicting TDEE in SB. The model selection procedure and predictive accuracy will be evaluated as described in the statistical methods for aim 1. Once the best BMR equation is identified, TDEE will be estimated as a weighted sum of estimated BMR and physical activity level. To handle the missing data issue, regression analysis will be conducted under multiple imputation under the missing-at-random (MAR) assumption [83]. Sensitivity analysis will be conducted to check for potential violation of the MAR assumption [84]. Simulation under MAR will be conducted to quantify the efficiency loss, defined as 1 – E (Ŷ [X*] – Y)2/E(Ŷ [X] – Y)^2^, where X* represents the observed data and *X* is the data with missingness. The exploratory aim based on the Block food and physical activity screeners will be analyzed through NutritionQuest. Descriptive analysis on frequency and duration of physical activities, sedentary activity, and youths’ dietary intake by food groups and servings will be presented. Raw accelerometer data are collected at 30 Hz and converted to counts through ActiGraph’s proprietary software (ActiLife; ActiGraph LLC). Analysis will include calibration of individualized activity counts aided by 20-minute ramped protocol data based on the perceived exertion of the youth and measured heart rate. The Block Kids Physical Activity Screener will complement the objective accelerometer data.

### Power Analysis

The power calculation was based on the increased coefficient of determination (*R*^2^) from a baseline simple linear model to a multivariate model that regresses body fat percentage on potential predictors (ie, alternate height, BMI, race, and age). Pilot studies from the literature suggest that the correlation between the body fat percentage measured using DLW and alternate height (arm span and recumbent length) is in the range of approximately 0.6 to 0.7 (*R*^2^=0.36-0.49). As we will use 10-fold cross-validation to construct our predictive models for 2 gender groups, a sample size of 94 (232/2 × 0.9 × 0.9) achieves 90% power to detect an increase of *R*^2^≥0.066 (0.078) attributed to 1 (2) independent variable(s) using an *F* test with a significance level of .05. The variables tested are adjusted for 1 independent variable in the simple linear model with an *R*^2^ of 0.36. We assume that 10% of the participants will not complete all measures based on pilot data. Because it is likely that we will include >2 additional variables in the final model, the proposed sample size will have sufficient power to detect a clinically meaningful increase in *R*^2^.

### Data Storage

Data are collected and stored using REDCap (Research Electronic Data Capture; Vanderbilt University) [85]. Through REDCap, individual levels of privilege and access can be granted to study team members based on need. This database provides menu-driven options and built-in checks and generates reports. All data are checked for accuracy through biweekly reports run by the lead study coordinator. Signed consent or assent forms are kept in a locked file cabinet in the office of each site’s lead investigator and are separate from deidentified study data. REDCap projects rely on a thorough study-specific data dictionary defined in an iterative self-documenting process by team members, with planning assistance from the administrator. The development and testing process results in a well-planned data collection strategy. The REDCap server is housed in a local data center of the lead study coordinator, and all web-based information transmission is encrypted with a 256-bit Secure Sockets Layer certificate. Data backups are performed nightly and stored in a separate location.

### Ethical Considerations

This human participant study was reviewed and approved by the Western IRB–Copernicus Group (20190134). Informed consent from a parent or legal guardian for all participants and assent for participating children aged ≥7 years is obtained by a trained staff member who has experience working with children. All documents are IRB approved and provided in English and Spanish based on the needs of the family. During the consent and assent process, it is reiterated that participation is voluntary and that there will be no consequences if they do not participate or complete the study. Signed consent and assent forms are kept in a locked file cabinet in each lead site investigator’s locked office, and a copy is provided to each family. The confidentiality and privacy of the participants’ information is protected by deidentifying the data and through the use of a secure, encrypted, and password-protected database for storage. Compensation for participation is provided using gift cards at 2 time points of the protocol. The monetary value reflects the participants’ time and effort required to complete the study protocol. To reduce family burden, an optional gift card of up to US $125 is available if needed to support travel costs to the study site or associated childcare costs. The amount is determined by each site based on their geographical region, distance to the study site, and associated travel costs.

## Results

This study was funded in July 2019, and training and study preparation occurred until February 2020. Data collection was initiated in March 2020 but then halted due to the COVID-19 outbreak, with a staggered and slow reintroduction initiated in September 2020 based on each organization’s policies and state mandates. During the COVID-19 outbreak, a 6-month midproject extension was provided by the funding agency that extended our study end date to November 30, 2024.

Recruitment challenges after the COVID-19 pandemic have persisted. While the incidence of COVID-19 has diminished across the United States, secondary effects are noted. This includes an increased turnover of research staff and a reduced number of patients being seen in the clinical setting for in-person appointments. At the beginning of the fourth year of the grant (December 2022), one of the 4 clinical sites withdrew secondary to increased challenges related to enrollment of participants. A new data collection site was added to replace the discontinued site. A subaward was successfully executed, and the site’s involvement was reviewed and approved by the IRB of record and at their local level. The team was onboarded with one-on-one training and set up with equipment required for the protocol. This process was completed over a 5-month period, with initiation of data collection occurring in late spring 2023.

As of April 2024, a total of 143 participants (female participants: n=76, 53.1%; male participants: n=67, 46.9%; Hispanic participants: n=52, 36.4%) have been enrolled. Recruitment and enrollment strategies are anticipated to continue until year 5, with a planned request for a no-cost extension year (December 2024-November 2025). The no-cost extension year will be used to complete analysis and initiate dissemination. Cleaning of data and preliminary analysis have begun and support a low rate of missing data and retention of participants throughout the 7-day protocol.

## Discussion

### Expected Findings

The aims of this study are to develop 2 algorithms for use in youth with SB in a clinical setting, one to model body fat and one to predict TDEE. In addition, the physical activity and dietary intake of the sample will be described. To increase the generalizability of the findings, the goal is to recruit a diverse sample of youths aged between 5 and 18 years who are diagnosed with myelomeningocele, the most severe type of SB. This population has an increased prevalence of overweight and obesity that influences their physical health, mobility, and level of independence.

Data cleaning and preliminary analysis have documented that there are limited missing data and a high retention of study participants who start the protocol. Current enrollment of 61.6% (143/232) of the proposed sample is on target to reach the goal of including 25.9% (60/232) of participants who self-identify as Hispanic.

Challenges encountered have primarily centered on COVID-19. Since this research project was funded, the COVID-19 pandemic drastically altered clinical programs and, thus, recruitment and data collection. COVID-19 affected the number of health care staff in all settings, resulting in overloads, reduced clinical activities, and unexpected vacancies in key clinic management and study personnel, thus hampering the implementation of the project in some sites. Although each of the clinical sites’ research compliance mechanisms differed, research at all sites was completely suspended for multiple months, with variability by site for resumption of research activities (recruitment and data collection). Each program or organization limited the number of research personnel allowed in clinical settings and the scope of recruitment activities upon resumption, which varied by site. In addition, telehealth drastically reduced clinic attendance in some settings. Finally, travel to sites for training and validation of data collection was challenged by the pandemic.

Although a 6-month extension of year 2 was obtained from the funding agency (National Institutes of Health/NICHD), recruitment remained variable and reduced at each of the study sites. Concurrent to the disruption of clinical processes for conducting research, families experienced challenges with school programs altering attendance and having multiple children at home, and more clinic participants lived a substantial distance from the clinic than was originally anticipated. Families voiced concerns about (1) childcare as clinical and research settings were allowing only 1 adult and no siblings to attend data collection, (2) the risk of public mass transit, (3) the cost of alternative transportation, and (4) difficulty traveling to the data collection site for early-morning appointments necessitated by the 6-hour preappointment fast. These concerns prompted amendments to the study protocol related to participant reimbursement and discontinuing data collection at 1 site and adding a new site.

Despite the unforeseen challenges, the strengths of this study are notable. We are the first to convene a sample of youth with SB to develop an algorithm that incorporates clinically available, cost-effective, but not yet validated measures of height or body composition to accurately model body fat percentage in this population and estimate TDEE. In addition, the purposive recruitment from clinics located in different regions allows for stratification by age and mobility status and provides representation of ethnicity while providing an adequately powered sample to test the aims. The use of DLW, a validated noninvasive measure, as the criterion for body fat percentage and TDEE increases the study’s rigor. The use of a DXA scan in a subgroup of the sample allows for a second comparison of body fat percentage and FFM between DLW and DXA, decreasing concerns of depending on a 2-compartment body water method in this unique population. Limitations include the lower-than-expected enrollment at the time of this publication. The team continues to strategize on methods to further recruitment and enrollment within the timeline of the grant. The findings will enhance the screening, prevention, and treatment of an abnormal weight by facilitating the accurate identification of the youths’ weight status and allow for recommendations of daily caloric needs to be provided for this population at a higher risk of having obesity. With the ability to accurately estimate body fat percentage, we can accurately categorize weight status and monitor weight trends longitudinally. Furthermore, our findings in youth with SB have the potential to impact outcomes for youth diagnosed with disabilities other than SB who experience similar challenges related to alterations in body composition or fat distribution or measurement challenges secondary to mobility issues or musculoskeletal problems. The NICHD research agenda that prioritized weight management for youth with disabilities was not limited to SB but included youth with a variety of intellectual and physical disabilities [28]. The successful execution of this study, methodological approach, and dissemination of the findings will shift current research and clinical practice paradigms as well as raise awareness for the need to conduct future studies in this area. Furthermore, the measures used in the proposed research will serve as a framework for use with children in other diagnostic categories and, thus, will make a major contribution to science.

Dissemination of the study findings will be a priority. Outcomes will be shared with national SB organizations (ie, Spina Bifida Association); the Centers for Disease Control and Prevention NSBPR national and international conferences focused on SB, weight management, and developmental disabilities; manuscripts; and individual and family organizations. Future directions for this work will be to test the application of these algorithms in youth with SB and expand this work to adults with SB. The outcomes of this study will also be implemented in weight management interventions.

### Conclusions

This innovative study will provide 2 clinically accessible and feasible algorithms related to body fat and TDEE. The lack of an accurate clinically available and cost-effective method to measure body fat and the inability to provide daily caloric recommendations obstruct the advancement of the science of weight management of youth with SB. These foundational deficiencies significantly impede successful prevention and treatment of obesity for this vulnerable population. The outcomes of this study will advance the science of weight management in individuals with disabilities, address national research priorities, and shift clinical practice paradigms.

## Abbreviations

BIA: bioelectrical impedance analysis
BMR: basal metabolic rate
DLW: doubly labeled water
DXA: dual x-ray absorptiometry
FFM: fat-free mass
FM: fat mass
IRB: institutional review board
MAR: missing-at-random
NICHD: Eunice Kennedy Shriver National Institute of Child Health and Human Development
NSBPR: National Spina Bifida Patient Registry
REDCap: Research Electronic Data Capture
SB: spina bifida
TBW: total body water
TD: typically developing
TDEE: total daily energy expenditure

## Data Availability

Data sharing is not applicable to this paper as no data sets were generated or analyzed during this study protocol.
